# A New Lavender (*Lavandula multifida* L.) Ecotype from Arid Tunisia, with Differential Essential Oil Composition and Higher Antimicrobial Potential

**DOI:** 10.3390/life13010103

**Published:** 2022-12-30

**Authors:** Mohanad Lateef Tofah, Khalil Mseddi, Omar K. Al-Abbasi, Ahmed Ben Yazid, Ahmed Khechine, Radhouane Gdoura, Lamia Khannous

**Affiliations:** 1 Research Laboratory of Environmental Toxicology Microbiology and Health (LR17ES06), Faculty of Sciences, Sfax University, BP 1171, Sfax 3000, Tunisia; 2 Research and Development Department, The State Company for Drugs Industry and Medical Appliances, Samarra 34010, Iraq; 3 Research Laboratory of Biodiversity & Ecophysiology of Arid Ecosystems, Faculty of Sciences, Sfax University, BP 1171, Sfax 3000, Tunisia

**Keywords:** *Lavandula multifida*, essential oil, chemical composition, antibacterial activity, habitat, morphological traits

## Abstract

The lavender *Lavandula multifida* L., a medicinal plant grown in arid regions of Tunisia, was recently considered an endangered species; thus, its habitats regressed to some difficult zones in terms of access, such as the watershed of Oued Agareb in central-eastern Tunisia. This species was recorded only in deep and narrow shady Wadi of the watershed and benefited from protection against overgrazing, erosion and sunlight. *L. multifida* was rarely observed in an open area, such as a plateau or large-bed valley. The plant’s metabolism is linked to its response to environmental conditions, which is of particular interest to understanding the components of the considered population of *L. multifida*. Consequently, biochemical and antimicrobial analyses have been evaluated. Using gas chromatography-mass spectrometry (GC-MS) analysis reveals that among the 58 compounds identified in *L. multifida* essential oil extracted from aboveground plant tissues, camphor was the major component (15.68%), followed by 1,8-cineole (14.14%) and alpha-pinene (13.82%). Moreover, it has been observed that *Escherichia coli* was more susceptible than *Staphylococcus aureus* to the antimicrobial properties of *L. multifida* essential oil, while in the case of camphor, *S. aureus* was more susceptible than *E. coli*. The protected population of *L. multifida* exhibits a distinctive vegetative development and growth cycle, resulting in specific secondary metabolites and distinguished antimicrobial activity.

## 1. Introduction

The family of *Lamiaceae* has been recognized for a long time as a source of many medicinal and aromatic species. The essential oil extracted from *Lamiaceae* species is proved by many studies to be of a high quality, and it was used by multiple industries, such as pharmaceutic and cosmetic, but especially in traditional medicine in many rural communities [1,2,3]. The commerce of plants and essential oils has increased considerably in both developed and developing countries [4]. In *Lamiaceae* species, the majority of the utilized material is derived from wild populations that have frequently been developed in disturbed environments [5,6,7]. Classical selection of medicinal plants (traditional selection) and biotechnological approaches for plant improvement (using biotechnological methods, such as tissue and organ culture, genetic transformation) were restricted to some renowned taxa (e.g., *Mentha* spp., *Lavandula* spp., *Salvia* spp., *Ocimum* spp., *Origanum* spp.). However, a severe decline and enormous loss, linked to both natural populations and many species belonging to the *Lamiaceae* family, have been reported during the last decade.

The harsh climate, the prolonged drought and the extension of agricultural fields by clearance, all aggravated by anthropogenic activities, such as overgrazing and urbanisation, are effective causes of the decline of wild *Lamiaceae* populations [8]. The harsh environmental conditions (climatic and edaphic factors), particularly in semi-arid and dry Mediterranean areas, have considerably contributed to increased genetic erosion. In North Africa, many species (such as *Thymus capitatus*, *Lavandula multifida* L., *Lavandula stoechas*, *Ajuga iva* and *Mentha* spp.) are endangered and grown in protected areas. Other species can only be found in old checklists [9,10].

The genus of *Lavandula* consists of about 51 wild and cultivated species grown in Mediterranean habitats [11]. Among them, *L. angustifolia* Mill. (Lavender), *L. latifolia* Medik., *L. stoechas* L., *L. dentata* L., *L. pinnata* Lundmark, *L. lanata* Boiss., *L. mairei* Humbert and *L. multifida* L. are the most appreciated and used in folk medicine and the cosmetic and pharmaceutic industries [12,13,14,15,16]. In Tunisia, the genus *Lavandula* includes three natural and popular species—*L. stoechas*, *L. dentata* and *L. multifida*—growing in semi-arid areas [17]. Le Floc’h (2008) [18] completed this list with the addition of another species, namely, *L. coronopifolia* Poiret in Lam. In the arid region of Tunisia, *Lavandula* has become rare and grows in very restricted protected zones, while sympatric populations are absent, as reported by Pottier-Alapetite [17]. In mid-eastern Tunisia, *L. multifida* is naturalised in habitats of semi-arid regions [19]. Furthermore, it has been adapted to some arid zones in southeastern regions of the country [17]. However, due to uncontrolled human harvesting of this species, as well as the overgrazing and regression of natural rangelands in the last decade, *L. multifida* has become an uncommon and endangered species [20].

*L. multifida* grows in open calcareous garrigues. The edaphic substratum is formed by the degradation of *Pinus halepensis* Mikll. and *Juniperus phoenicea* L. primary forests at an elevation ranging from 150 to 550 m [19]. Climatic traits of the native habitats are characterised by an annual rainfall between 350 and 450 mm, while the average annual temperature varies around 18 °C. The surrounding flora communities associated with *L. multifida* are usually composed of *Thymelaea hirsuta* L., *Rosmarinus officinalis* L., *Oryzopsis miliacea* L., *Rhus tripartita* D.C., *Hyparrhenia hirta* L., *Thymus capitatus* L., *Thymus algeriensis* L. and *Ebenus pinnata* Ait. In traditional medicine, *L. multifida* is used to treat many diseases, such as rheumatism [21], because of its inflammatory properties [22], as well as hypoglycemia [23]. In leaf extracts, a combination of luteolin 7-O-glycosides, hypolaetin-8-O-glycosides and isoscutellarin-8-O-glycosides and terpenoids (i.e., 15,16-dihydroxy-7,11-dioxopimar-8(9)-ene; 15S,16-dihydroxy-7-oxopimar-8(9)-ene) were investigated [24]. Ethanolic and aqueous extracts of the shoot system and aerial parts show high antioxidant activity [25]. Biochemical compounds were also identified in some populations of *L. multifida* grown in Tunisia [26]. Therefore, approximately 36 components, constituting 83.48% of the total oil, were identified in the essential oils extracted from the leaves of *L. multifida*. Carvacrol (31.81%), bisabolene (14.89%) and acrylic acid dodecyl ester (11.43%) were the major volatile components. These elements have also been identified as the main compounds of *L. multifida* selected from other Mediterranean habitats, including Morocco and Spain [27,28], where the main constituents were oxygenated monoterpenes (33.70%). Sesquiterpene hydrocarbons and b-bisabolene, with a percentage of 16.18% and 14.89%, respectively, of the oil are the most abundant components in this genus [26].

The habitat disturbance of *L. multifida* caused by overgrazing, uprooting of plants and cleaning activities has led to a regression of the species frequency. Such factors are linked to a disturbance, induced population fragmentation and large decrease in the size and number of individual plants, followed by a decrease of the green soil surface. The habitat’s degradation reduces the adaptive potential of *L. multifida* to environmental changes, causing it to possess more vulnerable populations [29].

The genetic variation within a population determines its ability to be maintained alive for long-term usage. As a result, analyzing genetic diversity within and across endangered species’ populations is critical to determine their future survival and to create improvement and conservation initiatives [30,31]. In Tunisia, because of *Lavandula*’s importance in traditional medicine and the pharmaceutic and cosmetic bio-industry, similar to most of the *Lamiaceae* species, *L. multifida* was over-exploited, leading to its becoming endangered [32,33]. Due to ongoing anthropogenic stresses, most *L. multifida* populations were fragmented and represented by scattered individuals in some protected locations [5]. Habitat fragmentation enhances population divergence and contributes to genetic diversity loss [30], while the maintenance of genetic variety is critical for population evolution and adaptability to environmental change [34]. As a result, understanding the current status of these endangered species (distribution area, regeneration capability, floral biology, genetic diversity) is critical to ensure their sensible usage.

The investigation of morphological heterogeneity, ecological adversity and population structure offers critical information for developing restorative initiatives and long-term conservation and improvement of the species [35,36]. The synthesis and accumulation of primary and secondary metabolites varies intensely amongst specimens of the same plant species cultivated under various environmental conditions and habitats [37,38,39]. Secondary metabolites that are stimulated by environmental factors (for example, water and salt stress in arid and semi-arid regions) act as a chemical interaction between the plant and its surroundings [38]. Plants and their environments interact largely through secondary metabolite production, which performs biological tasks, such as a flexible adaptive response to their habitats [40,41]. Therefore, the investigation of such discrepancies is extremely valuable for the chemical characterisation of individual plants of the same species collected from different habitats, especially in considering the different geographical origins of plant material [42,43]. These processes (genetic differences within a plant species) include either long-term acclimation or local adaptation, as well as seasonal variations in phenology, environmental fluctuations linked to biotic and abiotic factors and geographical disparities affecting diverse populations [44].

The aim of this research was the study of the morphological traits and ecological response of *Lavandula multifida* L. grown in the watershed of Agareb, one of the restricted areas of this species in mid-eastern Tunisia. Furthermore, secondary metabolites were analysed using GC-MS. Moreover, antibacterial properties were evaluated against two resistant strain bacteria, i.e., *Escherichia coli* and *Staphylococcus aureus*.

## 2. Materials and Methods

### 2.1. The Study Site

**Figure 1:**
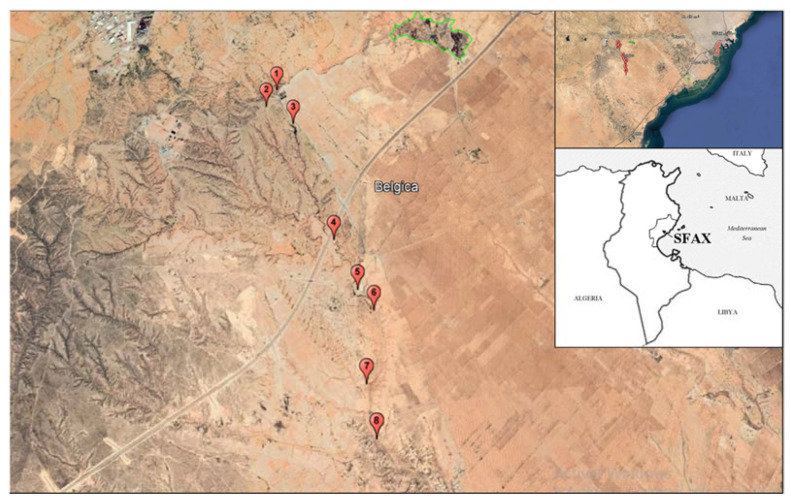
Study area, the watershed of Agareb in Sfax, Tunisia.

This study was carried out along 8 zones covering 29 stations in the Agareb region, which is considered a representative portion of the washout of Agareb (Figure 1). The Agareb region belongs to the Sahel of Sfax, located between latitudes 34°40′–34°50′ and longitudes 10°25′–10°45′. It is bounded from west to east by the plateaux of Jouaouda, Araba, El Gouna and Traka and the plains of Sfax, Ouled Ameur and Soualah.

The watershed of Oued Agareb, located on the southern coast of Sfax and subjected to an irregular climate, is characterized by shallow topography, a soft structure and a dense river network. These factors make the watershed fragile, thus triggering various forms of soil degradation. Washouts and deep wadi are the fundamental erosion structures that characterise this region. As narrow and deep structures, washouts can hold many sensitive species, such as *L. multifida*, that cannot resist the climatic severity outside the river area.

The climate of the study zone is characterised by an annual mean temperature ranging from 2 to 24 °C, with mean annual precipitation of 196 mm. During the study period, the mean air temperature of the cold season was 12.1 ± 0.5 °C and that of the hot season 26.4 ± 1.4 °C; the rainfall varied from 7 mm during the dry period to 75 mm in autumn and 65 mm in winter. The climate traits are characteristic of a Mediterranean Lower Arid climate with temperate winter. The soil is an alkaline sandy loam, with gypsum outcrops and crumbly caliches at a depth of 10–25 cm. The topography is characterised by hills traversed by many valleys and gully erosion. The flora is composed of a remaining degraded steppe of Alfa grass *Stipa tenacissima* L., hosting a sparse perennial vegetation mainly composed of *Artemisia herba-albaL., Artemisia campestris* L., *Thymelaea hirsuta* Endl. and *Retama raetam* (Forssk.) Webb.

Washouts of Agareb have special geomorphology characterized by a network of many valleys. The studied zone is located in the Sahel Sfax, the eastern Tunisian platform. The Agareb region has a monotonous topography showing mounds with a curvature of a large radius, separated by wide valleys, plateaus and plains, generally subsiding and occupied by sebkhas (i.e., smooth flat plains sometimes occupied after a rain by a shallow lake). The altitudes rarely exceed 200 m. In this area, the highest altitudes reach 141 m at Hamadet El Houch and 183 m at Ksar El Merdjine. The eight study areas are represented by wadis and their edges, as well as the plateaus located in proximity. The depth and width of the wadi beds are the fundamental differences between the different sites.

### 2.2. Plant Material and Morphological Traits

*Lavandula multifida* L. that belongs to the *Lamiaceae* family—known by the vernacular name fern leaf lavender and Halhal, as well as wild Khezama, a local name—is an herbaceous perennial, highly aromatic, ever-blooming plant native to Southern Italy, Spain and Northern Africa (Morocco, Algeria, Tunisia and Libya) and frequently connected with open, disturbed regions and settlement.

The specimens were collected from eight sites along the studied washouts during the period 2019–2020. Dr. Khalil Mseddi (Botanic section) from the department of Biology, Faculty of Science, University of Sfax, has identified the plant at the species level. A voucher specimen (AGT 001) was deposited in the herbarium of the Department of Biology, University of Sfax, Tunisia.

The geographic coordinates were estimated by GPS. Ten morphological characteristics, i.e., root length (cm), number of root ramifications, root diameter (cm), stem length (cm), number of stem ramifications, leaf number per stem, spike length (cm), number of flowers per spike and flower length (cm), were measured in the collected samples.

### 2.3. Extraction of Essential Oil

Essential oils, also called volatile odoriferous oils, are aromatic oily liquids extracted from different parts of plants, such as the leaves, peels, barks, flowers, buds and seeds. They can be extracted from plant materials by several methods, including steam distillation, a method that has been widely used, especially for commercial-scale production. The composition of each essential oil depends not only on the family, but also on the part of the plant from which it is extracted, and sometimes on the soil where the plant grows, or even on the time of the harvest. Therefore, gas chromatography is necessary to characterize an essential oil.

Extraction of essential oil from the collected plant material was performed, as previously described by Baj et al. [45]. Briefly, 50 g of dried aerial parts of *L. multifida* collected from deep wadi in semi-arid Tunisia was placed into a round flask, and 400 mL of distilled water was added. The Hydro distillation was performed by a Clevenger apparatus (Glassco) for 3 h, according to the above-mentioned method. Extracted essential oils were collected in vials and stored at 4 °C until GC-MS analysis.

### 2.4. Chemical Analysis

The analysis was carried out using a gas chromatograph-mass spectrometer, GC-MS QP 2018, Shimadzu, equipped with a split-splitless injector and a ZB-5MS capillary column (30 m × 0.25 mm; 0.25 µm film).

### 2.5. Chromatographic Conditions

The column’s initial temperature was 45 °C, which was held for 2 min before ramping up to 280 °C at a rate of 5 °C per minute and then remaining steady for 5 min. The injector’s temperature was 265 °C. Helium (the carrier gas) flowed at a rate of 1 mL/min. The following conditions were applied for acquiring mass spectra: (instrument current) ionization voltage, 70 eV; ion source, 200 °C; filament emission current, 60 mA. Automatic essential oil injections were performed at a 1:15 split ratio, and the injection of the essential oil was at (1 µL,). The range of the mass spectrometric detector, which was in scan mode, was 35 to 550 *m/z*. By comparing mass spectra and retention indices in a high-quality Shimadzu mass spectral library, essential oils were identified.

### 2.6. Tested Microorganisms

*Lavandula multifida* L. is a member of the genus of Lavender that belongs to the mint family (*Lamiaceae*). These aromatic plants produce valuable and essential oils (EOs) in the manufacture of medicines, perfumes, cosmetics and food, as it has antioxidant properties and biological properties against the growth of many microorganisms, especially *Candida albicans*, *Staphylococcus aureus* and *Escherichia coli*. It is also widely used in folk medicine as an antispasm.

More than 95% of urinary tract infections are caused by *E. coli*, which is the most pathogenic organism. However, many other bacteria can also cause pathogenicity, such as *Staphylococcus*, *Pseudomonas*, *Proteus*, *Klebsiella* and *Neisseria*. Antibiotic resistance among *E. coli* has been recorded internationally, and growing resistance rates among *E. coli* are of increasing concern in both developed and developing countries.

The various components of plant extracts play an important role as antibacterial agents, as it has been suggested that vegetable oils act through the lipophilic part that interacts with the fatty parts of cell membranes. In recent years, multi-antibiotic resistance has arisen due to the indiscriminate use of antibiotics; hence, this problem requires efforts to find effective treatments by searching for effective materials against the microorganisms that cause these diseases. In addition, synthetic antibiotics are powerful and life-saving medicines. Yet, they do more harm than good when not used correctly; thus, there is also a need to develop alternative antimicrobial medicines to treat infectious diseases from other sources. The antibacterial properties of the essential oils of *Lavandula multifida* were evaluated against these two resistant bacteria strains, i.e., *Escherichia coli* and *Staphylococcus aureus*.

### 2.7. Antimicrobial Activity

The antimicrobial activity of the *L. multifida* essential oil was tested against two strains of pathogenic bacteria: *Escherichia coli* ATCC 8739 and *Staphylococcus aureus* ATCC 6583. The two microorganisms were derived from the culture collection of the QC department of the State company for the drugs industry and medical Appliance/Samarra-Iraq. The agar well diffusion method was employed for the determination of the antimicrobial activities of the tested diluted essential oil, as described by Oumzil et al. [46] with some modifications. Briefly, the test was performed in sterile Petri dishes containing Muller Hinton agar. A total of 6 wells (6 mm in diameter) were impregnated with 100 μL of oil and were placed on the Petri plates previously inoculated with a microbial suspension. The suspension of bacteria was obtained from 18 h cultures (one microorganism per Petri plate). All Petri plates were incubated at 37 °C for 24 h. The diameters of inhibition zones were measured in millimeters.

For determining the minimum inhibitory concentration (MIC) values of the essential oil and camphor material against the two bacterial strains, we tested 5 serial concentrations, ranging from 5 to 2.5, 1.25, 0.625 and 0.312 μg/mL. This method was done according to the CLSI protocol (CLSI, 2017), with some modifications as previously described by Sakkas et al. [47]. The dilutions of oil were prepared in Mueller–Hinton broth (MHB, Oxoid Ltd., Basingstoke, UK). Tween 20 (Thermo Fisher Scientific, Waltham, MA, USA) was used as a solubilizer at a concentration of 0.5% (*v*/*v*). Bacterial inoculum measured at turbidity of 0.5 McFarland (1–1.5 × 108 CFU/mL) was transferred into MHB to obtain a bacterial count of 5 × 105 CFU/mL. After incubation for 24 h at 37 °C, an appropriate amount of 10 µL was added to MHB and incubated for 24 h at 37 °C. The visible method was used for the determination of MIC value. The MIC is defined as the lowest concentration of an antimicrobial agent (EO) that prevents the visible growth of a microorganism in a broth dilution susceptibility test.

## 3. Results

### 3.1. Ecological Context and Distribution

Due to rangeland overgrazing and climate severity, *L. multifida* has become a rare species in the watershed of Oued Agareb. This work shows that the studied population exhibited shade preference strictly related to deep and narrow Wadi, which were the result of channel erosion along gully beds, whereas this species was absent in the open area of the plateau and hill zones (Table 1). This might be attributed to overgrazing and strong winter winds.

However, it was seen as a random individual species only in the protected park of Algonna (created near the study zone), associated with some phanerophytes species, such as *Retama reatam* that can create a protective micro-environment and habitat and minimize sunlight effect. Hydraulic networks crossed by washouts were the fundamental erosion structures that can be seen in this region, which can support the growth of many sensitive species, such as *Lavandula multifida*, that cannot resist or tolerate the climatic severity outside the valley.

### 3.2. Communities Associated with L. multifida

The growth of *L. multifida* can be associated with that of numerous neighbor species (Table 2). However, it was observed that the species *Retama raetam, Lycium shawii*, *Thymelaea hirsuta*, *Cenchrus ciliaris*, *Rhus tripartita* and *Aspargus albus* were the nearest. These species can offer protection against both overgrazing and climate severity via their upright stem development and/or spiny leaves and stems.

Some other species can be associated in a distance of one to two meters around *L. multifida*. These species do not have any direct contact among aerial shoots, but they may communicate with their underground parts (rhizomes and roots). The most common species were *Trigonella stellata*, *Stipa capensis*, *Rhanterium suaveolens*, *Lygeum spartum*, *Artemesia* *herba-alba*, *Gymnocarpos decander*, *Asparagus albus* and *Hyparrhenia hirta.* Species such as *Hammada scoparia*, *Artemisia campestris*, *Anabasis oropediorum*, *Ajuga iva*, *Peganum harmala*, *Periploca laevigata*, *Teucrium polium*, *Allium roseum*, *Fagonia cretica*, *Asparagus stipularis* and *Atractylis serratuloides* were also seen in the same habitats as *L. multifida*.

### 3.3. Phenology and Life Cycle

According to the Raunkiaer classification [48], *Lavandula multifida* is a Chamaephyte (dwarf shrub) plant, exhibiting stems growing near the ground, and it bears dormant, hibernating buds that grow close to the ground, approximately 25 cm above the soil surface. The advantage of being close to the soil surface is that the buds can be protected from many negative environmental impacts. As a result, the chamaephyte habitat is more widespread in stressed niches, with prolonged periods of drought and severe herbivore grazing pressures.

**Figure 2:**
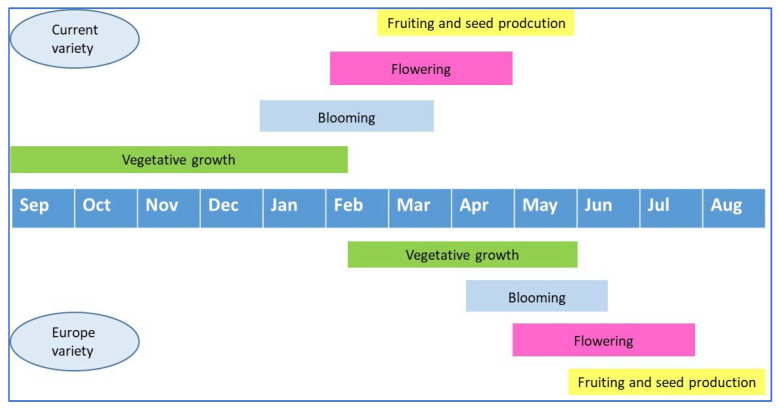
Life cycle of the Europe and the current variety of *L. multifida*.

Current varieties of *L. multifida* have an autumnal–winter life cycle, which is different than the European variety (spring–summer cycle) [49]. Indeed, this species grows rapidly and with high density just after the first autumnal rain (September–October), reaching maximum development in winter (Figure 2). *L. multifida* blossoms between late autumn and winter (i.e., from September to January). The development of fruits and seed dispersal are both completed in the beginning of spring. During the dry summer with elevated temperatures, the plants of *L. multifida* lose the majority of their leaves and become dry, woody shrubs bearing hidden buds during the unfavorable season.

### 3.4. Morphological Traits

**Figure 3:**
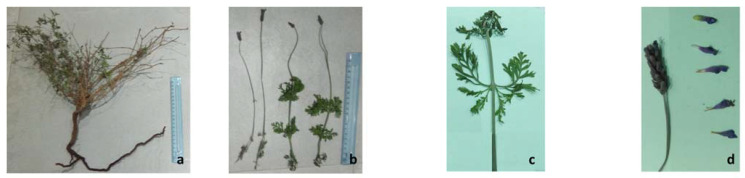
Views of vegetative and reproductive parts of *Lavandula multifida*. (**a**) The whole plant (woody structure of shoot and root systems), (**b**) External leafy and internal naked shoot, (**c**) Deeply lobed leaves; (**d**) Inflorescence and flowers.

The mean values of the considered parameters were calculated on collected samples from the study zones, and the results are presented in Table 3. The population of *L. multifida* in the Agareb region typically grows up to 58.7 cm height on straight stems with deeply lobed, lacy, silver-green leaves (Figure 3). It has been published that *L. multifida* is a perennial species (Missouri Botanical Garden, *L. multifida*); however, it is actually a sub-shrub because it develops woody stems over time in areas where it is winter resistant. Its roots can reach 41 cm in length and cross with other roots of neighbor plants, such as *Cenchrus ciliaris* and *Retama reatam*.

### 3.5. Composition of Essential Oil

Gas chromatography-mass spectrometry (GC-MS) analysis of the essential oil obtained from the leaves of *L. multifida* showed that among the 58 identified compounds, camphor was the major component (15.68%), followed by 1,8-cineole (14.14%) and alpha-pinene (13.82%) (Table 4). These major compounds are followed by Linalool L (9%), Linalyl anthranilate (6.08%), Borneol L (6.04%) and Delta 3-Carene (5.43%).

### 3.6. Minimum Inhibitory Concentration (MIC)

The results of the minimum inhibitory concentration of the *L. multifida* oil and synthetic camphor are shown in Table 5. A difference in the minimum inhibitory concentration between the extracted lavender oil and camphor was detected, but there was a similarity in their inhibitory concentrations on each one of the two bacteria.

### 3.7. Microbiological Activity

**Figure 4:**
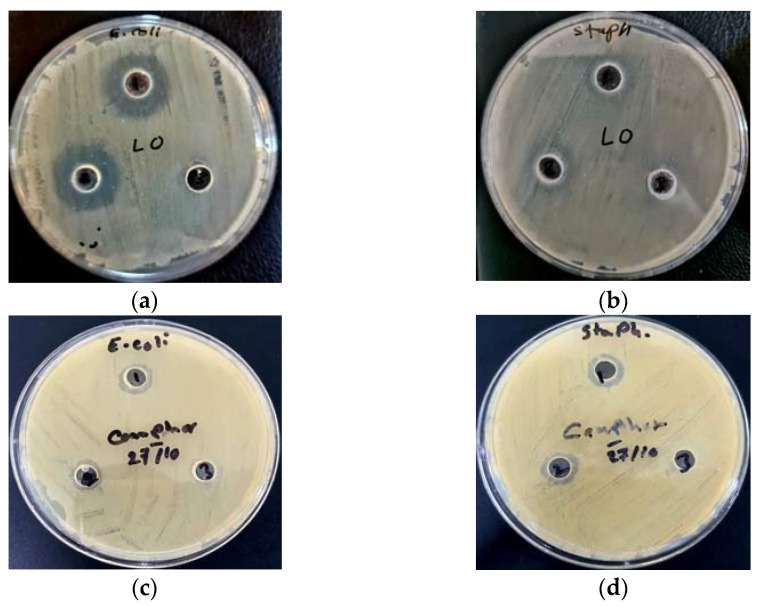
The biological activities of volatile (**a**,**b**) oil and camphor (**c**,**d**) from aerial organs of *L. multifida* against *E. coli* and *S. aureus* strains.

Measurement of the antibacterial activity of *L. multifida* essential oil against *E. coli* and *S. aureus* was made in comparison with the antibacterial activity of camphor as a major compound, in terms of concentration in GC analysis. According to the results (Table 6; Figure 4), a *L. multifida* oil concentration of 1.25 μg/mL possessed the highest inhibitory diameter (22.6 mm) on *E. coli*, compared to the lowest inhibitory diameter in *S. aureus* (17.8 mm), while the concentration of 0.625 μg/mL possessed the highest inhibitory diameter (21.3 mm) on *E. coli*, in comparison to the lower inhibition diameter on *S. aureus* (15.6 mm). In the case of camphor, the highest inhibition diameter (14.1 mm) was recorded on *S. aureus* at a concentration of 2.5 μg/mL of extract, in comparison to the lowest inhibition diameter recorded on *E. coli* (13.2 mm).

## 4. Discussion

Various studies have reported the genetic diversity [19,20,50] and the biochemical compositions [26,51] of different populations of *Lavandula multifida*. However, no previous works linking the morphological and phenological traits to the secondary metabolism and the essential oil composition have been reported. The current study shows that the morphological traits and habitat proprieties strongly affect the chemical composition of *L. multifida* essential oil and its antimicrobial activities.

In the current study, we found that the roots of *L. multifida* are 40 cm in length and 1 cm in diameter, a structure that may serve as either a drought tolerance or avoidance mechanism. The parameters of the shoots were quite different than those reported by Upson and Jury (2002) [52] for varieties of this species grown in Morocco. In addition, genetic analysis reported the high diversity in *L. multifida* populations from Tunisia [19,50]. 

The studied population starts its life cycle at the beginning of autumn with the first rain and rises to a maximum development in the end of the autumn and mid-winter. The blooming period is extended during winter, and fruiting is completed during spring. This life cycle is characteristic of the south Mediterranean population, i.e., North Africa regions [52]. However, in North Mediterranean regions (Spain and Italy), the flowering period begins in spring and is completed in summer [53].

Due to anthropogenic effects on the natural environment, *L. multifida* populations appear to be diminished and dispersed in North Africa (Morocco, Algeria, Tunisia, Egypt), Southern Spain and Portugal, Sicily, and Southern Italy [54,55,56]. Numerous of the above-mentioned studies have reported that biometric variables, such as morphological and reproductive parameters, display very different values, testifying to the high interspecific diversity [52]. Further, the secondary metabolism was related to environmental conditions [57].

In this study, 58 compounds were identified, and the major components were camphor (15.68%), 1,8-cineole (14.15%) and alpha-pinene (13.82%). Comparison with the previous study reported by Messaoud et al. [58] shows a different composition for the variety collected from the National Park of Bouhedma (Sidi Bouzid, Tunisia). The main components of *L. multifida* oil are carvacrol (65.1%) and beta-bisabolene (24.7%). In other work reported by Msaada et al. [51] on the variety collected from the Grombalia greenhouse (North-Eastern Tunisia), *L. multifida* oil was characterized by the predominance of linalool (50.05%), camphene (10.06%), linalyl acetate (7.30%), α-thujene (3.83%), bornyl acetate (3.03%), β-caryophellene (2.13%), nerol (2.01%) and terpinolene (2.05%). Chograni et al. [26] reported a different essential oil composition when they studied twelve populations of *L. multifida* collected from different bioclimatic areas in Tunisia. The major components at the species level were carvacrol (31.81%), beta-bisabolene (14.89%) and acrylic acid dodecyl ester (11.43%).

In North Africa countries, Saadi et al. (2016) [59] have reported that the major components of *L. multifida* essential oil growing in Algeria was carvacrol (61.73%) in inflorescence and (50.92%) in leaves, followed by linalool (5.69%) in inflorescence and anethole (17.37%) in leaves. Znini et al. (2019) [60] identified carvacroal (65.6%) as the major compound, followed by spathulenol (8.6%) and Para-Gymen-8-ol, in *L. multifida* grown in Morocco. These differences in essential oil composition are probably linked to micro-environmental conditions, such as soil, light, temperature and humidity.

The diversity of the composition of the volatile oil obtained from different accessions of *L. multifida* can be explicated by endogenous (plant varieties, vegetative state, organ tested) and exogenous factors, such as climatic features, soil characteristics and seasons [61].

The biological activity of *L. multifida* essential oil against gram-negative and gram-positive bacteria may be due to the effect of its chemical components, especially linalool and comphor. Our results are consistent with previous investigations, which evaluated the MIC of linalool for *L. augustifolia* (52.59%), exhibiting biological activity against gram-negative and gram-positive bacteria at a concentration of 1.25 μg/mL [62]. However, Jianu et al. (2013) [63] published the effect of *L. augustifolia* essential oil with a high content of camphor against *S. aureus* and *E. coli.* In another study [64], it was shown that *L. hybrida* essential oil containing linalool (32.8%) has inhibitory activity against *S. aureus,* while the opposite holds true for *E. coli*. Moreover, it has been published that the essential oil of *Lavandula stoechas* possesses inhibitory activity against both *E. coli* and *S. aureus* at MIC limits, which range between 0.16 and11.9 μg/mL [65].

Based on our findings, the essential oil of *L. multifida* was found to be more active against the used bacterial strains than camphor. As has been reported by Xu et al. (2005) [66], camphor’s mode of action on the development of microorganisms involves disruption of the phospholipid bilayer structure, interaction with membrane enzymes and proteins, and acting as a proton exchanger. The higher antibacterial activity of *L. multifida* essential oil against *S. aureus* and *E. coli* in comparison with camphor could be explained by the synergistic action of the components of this essential oil extract, especially the linalool and camphor content.

The extracted lavender oil from aerial plant tissues of *L. multifida* was biologically effective on gram-negative and gram-positive bacteria because it contained multiple biologically active substances, including camphor, which, in turn, exhibited an inhibitory effect against bacteria. The spread of *L. multifida* within the biodiversity of naturally growing plants in the valley seems to play a key role in the diversity of the extracted compounds.

## Figures and Tables

**Table 1:** Coordinates and geomorphological traits of *L. multifida* habitats. Presence of *L. multifida*: −: absent; +: low density; ++: high density; +++: very high density.

Zone	Station	Latitude	Longitude	Altitude m	Presence of *L. multifida*	Geomorphology
1	1	34°42′49.13″ N	10°32′45.10″ E	116	+	Bed of wadi
	2	34°42′53.67″ N	10°32′43.56″ E	100	+++	Deep narrow wadi
	3	34°42′54.22″ N	10°32′45.87″ E	104	−	Plateau
2	4	34°42′38.95″ N	10°32′38.59″ E	117	++	Beginning of wadi
	5	34°42′36.17″ N	10°32′40.14″ E	115	−	Plateau
	6	34°42′36.04″ N	10°32′37.23″ E	114	+++	Wadi dam
	7	34°42′35.54″ N	10°32′36.39″ E	116	−	Plateau
3	8	34°42′31.50″ N	10°32′58.09″ E	105	−	High slope wadi
	9	34°42′30.98″ N	10°32′57.42″ E	104	+	Bed of wadi
	10	34°42′33.11″ N	10°32′54.54″ E	107	++	Wadi narrow bed
	11	34°42′36.11″ N	10°32′51.20″ E	110	+++	Wadi dam
	12	34°42′32.54″ N	10°32′52.21″ E	109	−	Plateau
4	13	34°41′34.87″ N	10°33′23.66″ E	75	++	Deep narrow wadi
	14	34°41′32.55″ N	10°33′24.75″ E	71	+	Wadi narrow bed
	15	34°41′31.47″ N	10°33′22.64″ E	69	−	Open large wadi
	16	34°41′29.95″ N	10°33′25.06″ E	69	−	Open large wadi
	17	34°41′35.01″ N	10°33′19.67″ E	72	−	Open large
	18	34°41′31.75″ N	10°33′15.94″ E	70	−	Large bed wadi
5	19	34°41′12.72″ N	10°33′35.78″ E	64	−	Open wadi
	20	34°41′6.17″ N	10°33′37.02″ E	63	−	Large bed wadi
	21	34°41′5.80″ N	10°33′41.65″ E	63	+	Wadi deep bed
	22	34°41′6.83″ N	10°33′42.10″ E	63	+	Wadi deep bed
	23	34°41′8.62″ N	10°33′41.94″ E	63	+++	Wadi deep narrow bed
	24	34°41′10.07″ N	10°33′42.67″ E	65	+++	Wadi deep narrow bed
6	25	34°41′8.96″ N	10°33′43.09″ E	64	−	Plateau near Wadi
	26	34°41′3.55″ N	10°33′45.25″ E	63	−	Large wadi bed
7	27	34°40′59.58″ N	10°33′46.70″ E	62	−	Plateau
	28	34°40′22.39″ N	10°33′43.42″ E	53	−	Finish of Wadi
8	29	34°40′1.07″ N	10°33′47.33″ E	49	−	Tree culture

**Table 2:** Plant communities associated with *Lavandula multifida* in different study zones. Presence of *L. multifida*: −: absent; +: low density; ++: high density; +++: very high density.

Presence of *L.m.*	++	+++	++	+	−	+++	−	−
Geomorphology in Wadi	Wadi Up-Stream	Deep Narrow Wadi	Deep Bed	Large Bed	High Slope Wadi	Wadi Dam	Wadi Down-Stream	Plateau
*Asparagus albus* L.	+	+	+			+		+
*Agyga iva* (L.) Scherb.				+				+
*Allium roseum* L.	+			+	+			
*Anabasis oropediorum* Maire								+
*Artemisia campestris* L.			+	+				+
*Artemisia herba-alba Asso*		+	+	+				+
*Asparagus stipularis* Forssk			+	+				+
*Atractylis serratuloides* Sieber								+
*Cenchrus ciliaris* L.		+	+			+		
*Fagonia cretica* L.								+
*Filago germanica* L.								+
*Gymnocarpos decander* Forssk.		+						
*Hyparrhenia hirta* (L.) Stapf						+		+
*Lavandula multifida* L.			+	+				
*Lycium shawii* Roem. & Schult.		+				+		
*Lygeum spartum* L.			+					
*Peganum harmala* L.								+
*Periploca laevigata Aiton*								
*Deverra tortuosa* (Desf.) DC.						+		+
*Plantago albicans* L.				+				+
*Retama raetam (Forssk.) Webb*			+	+		+		+
*Rhanterium suaveolens* Desf.			+					
*Rhus tripartita* (Ucria)			+					
*Stipa tenacissima* L.								+
*Teucrium polium* L.				+				
*Thymelaea hirsuta* (L.) Endl.				+				
*Trigonella stellata* Forssk	+							

**Table 3:** Morphological traits of the studied population of *Lavandula multifida* grown in the Agraeb region, a semi-arid area in Tunisia.

Parameters	Description	Average Value
Roots	Woody, strong and deep. Length going from 17 to 41cm	24.5 ± 4.5 cm
Root branching	1–3 long ramifications	2.5 ± 0.5 ramifications
Root diameter	0.8–1.0 cm	1 ± 0.2 cm
Stem length	45–72 cm	58.7 ± 18.3 cm
Leaves	Deeply lobed, lacy, silver-green; usually twice pinnately divided into narrow segments	2.0 ± 0.5 cm long
Phyllotaxy	2 and sometimes 3 whorl/stem	2.5 ± 0.5 whorl/stem
Number leaves/whorl	Leaves disposed in 4–5/whorls	4.5 ± 0.5 leaves/whorl
Spike (inflorescence)	Dense with 4 rows, with 7–10 flowers.Position in terminal spikes (5 cm long), apical stems rising above the foliage to 15–45 cm tall	4 ± 1 cm long 36 ± 3.5 flowers/spike 38.5 ± 7.5 cm leafless stems long
Flowers		length; 0.8 ± 0.05 cm

**Table 4:** The composition (% *w*/*w*) of the essential oil of *Lavandula multifida*.

No	Compounds	% *w*/*w*	Substance, Group	RT (min)	Kovats Retention Index
1	Tricyclene	0.11	monoterpene	6.361	924
2	Alpha-Pinene	13.82	terpene	6.786	939
3	Camphene	2.51	monoterpene	7.16	954
4	1,3,5-Cycloheptatriene, 3,7,7-trimethyl	0.13	annulene	7.809	970
5	beta-Pinene	0.99	monoterpene	8.104	981
6	beta-Myrcene	0.40	monoterpene	8.701	989
7	Delta 3-Carene	5.43	monoterpene	9.5	1006
8	alpha –Terpinene	0.12	monoterpene	9.671	1018
9	*p*-cymene	0.58	monoterpene	9.811	1027
10	1,8-Cineole (Eucalyptol)	14.15	monoterpenoid	10.149	1034
11	Limonene	1.20	monoterpene	10.206	1032
12	trans-beta-Ocimene	0.09	monoterpenes	10.538	1037
13	1,3,7-Octatriene, 3,7-dimethyl-	0.14	monoterpenes	10.994	1046
14	gamma-Terpinene	0.10	monoterpene	11.373	1061
15	cis-Linaloloxide	0.11	furanoid	11.731	1066
16	Alpha-Terpinolene	0.13	p-menthadiene	12.644	1087
17	Linalool L	9.00	monoterpenoid	13.153	1100
18	Camphor	15.68	cyclic monoterpene ketone	14.44	1153
19	Borneol L	6.04	Terpene	15.758	1177
20	Lavandulol	0.17	monoterpene alcohol	15.97	1162
21	4-Terpineol	0.31	terpineol	16.287	1184
22	Alpha Terpineol	2.40	isomeric monoterpenoids	16.847	1198
23	Hexyl butanoate	0.12	fatty acid ester	17.174	1190
24	Linalyl anthranilate	6.08	linalyl ester	20.464	2157
25	l-Bornyl acetate	1.84	monoterpenoid and an acetate ester	21.564	1302
26	Lavandulyl Acetate	0.80	acetate ester	22.01	1283
27	Camphene	0.60	monoterpene	24.469	954
28	Neryl Acetate	0.22	acetate ester	25.118	1355
29	alpha-Copaene	0.50	hydrocarbon	26.01	1375
30	alpha-Gurjunene	0.77	carbotricyclic	27.271	1475
31	trans-Caryophyllene	2.28	sesquiterpene	27.525	1422
32	alpha-Humulene	1.71	monocyclic sesquiterpene	28.698	1458
33	trans-Caryophyllene	0.18	sesquiterpene	28.978	1422
34	γ-Muurolene	0.11	sesquiterpene	29.507	1476
35	Germacrene D	0.07	sesquiterpenes	29.622	1492
36	(+)-Epi-bicyclosesquiphellandrene	0.15	sesquiterpenoid	29.788	1489
37	alpha-selinene	0.07	sesquiterpenes	30.14	1521
38	(+)-Aromadendrene	0.09	sesquiterpenoid	30.255	1447
39	γ-Cadinene	1.77	sesquiterpenes	30.722	1513
40	Cis-Calamenene	0.42	sesquiterpene	30.831	1538
41	(+)-delta-Cadinene	1.11	sesquiterpenes	31.048	1531
42	Alpha-Calacorene	0.19	sesquiterpenoid	31.417	1538
43	Caryophyllene oxide	0.27	sesquiterpenoid	32.59	1586
44	alpha-Caryophyllene	0.14	sesquiterpene	33.062	1422
45	o-Menth-8-ene	0.33	homopolymer	33.357	1488
46	Naphthalene, 1,2,3,4,6,8a-hexahydro-1-isopropyl-4,7-dimethyl	0.74	sesquiterpenoid	33.726	1539
47	Dehydroxy-isocalamendiol	0.30	Sesquiterpene	34.022	1645
48	Humulane-1,6-dien-3-ol	0.46	Sesquiterpenoids	34.146	1606
49	alpha-Cadinol	2.27	sesquiterpenoid	34.452	1660
50	Ledol	0.30	sesquiterpenoid	34.535	1563
51	Benzonaphthofuran	0.36	Hetero-Polycyclic	35.267	2089
52	alpha-Bisabolol	0.64	monocyclic sesquiterpene alcohol	35.729	1684
53	2-Methoxy-10H-phenothiazine	0.09	phenothiazines	37.285	2542
54	Isothujol	0.09	bicyclic monoterpenoids	37.405	1157
55	Tridecane	0.07	alkane	38.043	1299
56	Tritetracontane	0.07	phenol	38.754	4300
57	Octadecane	0.07	alkane hydrocarbon	39.511	1800
58	Hexatriacontane	0.09	alkane	39.833	3600
Total		98.98			

**Table 5:** Minimum Inhibitory Concentration (MIC) of *L. multifida* oil and camphor on *E. coli* and *S. aureus*.

Strain	Extracts	Concentrations (μg/mL)				
		5	2.5	1.25	0.625	0.312
*E. coli*	*L. multifida* oil	+	+	+	+	-
	Camphor	+	+	+	-	-
*S. aureus*	*L. multifida* oil	+	+	+	+	-
	Camphor	+	+	+	-	-

**Table 6:** Biological activity of *Lavandula multifida* oil and Camphor against two bacteria.

	Essential Oil (μg/mL)			Camphor (μg/mL)		
	1.25	0.625	1.25	0.625	1.25	0.625
*E. coli*	22.6	21.3	-	13.2	10.6	-
*S. aureus*	17.8	15.6	-	14.1	12.2	-

## Data Availability

All data generated by this project are included in the paper.
